# A Clinical‐Radiomics Nomogram Based on Pretreatment Magnetic Resonance Imaging Predicting Tumor Residual at the End of Radiotherapy in Patients With Nasopharyngeal Carcinoma

**DOI:** 10.1155/ijbi/7443570

**Published:** 2026-08-07

**Authors:** Pingyan Liao, Min Zeng, Haitao Sun, Rongliang Xi, Guosen Huang

**Affiliations:** ^1^ Department of Radiation Oncology, Zhongshan Hospital of Traditional Chinese Medicine Affiliated to Guangzhou University of Traditional Chinese Medicine (Zhongshan Hospital of Traditional Chinese Medicine), Zhongshan, Guangdong, China; ^2^ School of Biomedical Engineering, Southern Medical University, Guangzhou, Guangdong, China, fimmu.com; ^3^ Medical Imaging Department, Zhongshan Hospital of Traditional Chinese Medicine Affiliated to Guangzhou University of Traditional Chinese Medicine (Zhongshan Hospital of Traditional Chinese Medicine), Zhongshan, Guangdong, China; ^4^ Guangzhou Aiweidi Biotechnology Co. Ltd., Guangzhou, Guangdong, China

## Abstract

**Background:**

The TNM staging system is a main tool for treatment stratification and prognosis prediction of nasopharyngeal carcinoma (NPC). However, patients with identical clinical stages often show different tumor regression patterns.

**Purpose:**

This study is aimed at constructing a nomogram model integrating MRI and clinical variables to predict primary tumor residual status at the completion of radiotherapy for NPC cases.

**Study Type:**

The present study is a retrospective design.

**Population:**

Two hundred NPC patients who accepted radical radiotherapy in the present hospital from December 2017 to March 2023 were systematically reviewed. The selected 200 NPC cases were divided into the training cohort (*n* = 160) and validation cohort (*n* = 40).

**Field Strength/Sequence:**

1.5T MRI scan, contrast‐enhanced T1‐weighted (CE‐T1WI) and T2‐weighted (T2WI) sequences.

**Assessment:**

For the training cohort, the LASSO with logistic regression was conducted to choose important features and construct a radiomics model. The clinical variables of patients were selected on the basis of statistical analysis and previous research. The nomogram was developed by combining the clinical factors with the radiomics features.

**Statistical Tests:**

Statistical analyses were conducted using R software for correlation analysis, chi‐squared test, multivariable regression analysis, Mann–Whitney *U* test, ROC curve analysis, and DeLong test. Significant level was *p* < 0.05.

**Results:**

Up to 109 radiomics characteristics were extracted, from which seven stable characteristics were selected to generate the Rad‐score. According to the regression result, T stage can be regarded as an independent predictor of primary tumor residual status. A nomogram combining the Rad‐score and clinical variables was established and proved, yielding an AUC of 0.836 (95% CI: 0.766–0.889, *p* < 0.001) in the training set and 0.843 (*p* < 0.001) in the validation set, respectively. The DeLong test indicates that the nomogram model is superior to both the individual clinical variables and the Rad‐score alone. The calibration curve and decision curve analyses proved the validity and clinical utility of the proposed model.

**Data Conclusion:**

A clinical‐radiomics nomogram has good efficacy in predicting primary tumor residual after radiotherapy, providing individual treatment strategies for NPC cases.

## 1. Introduction

The nasopharyngeal carcinoma (NPC) is a typical malignancy in southern China, but rare in Europe and the United States [1–3]. Radiotherapy is the cornerstone of the treatment of NPC. Due to the difference in the sensitivity of tumor radiotherapy, some patients experience good tumor regression after radiotherapy, whereas others exhibit tumor residual. Previous studies have shown that patients with residual tumor after radiotherapy have a worse prognosis [4–6]. Therefore, it is very important to achieve early and accurate identification of tumor residual before treatment, and for some patients with high residual risk, more aggressive treatment strategies can be adopted.

The TNM staging system has traditionally guided treatment stratification and prognosis prediction in NPC. Nevertheless, patients at the same clinical stage frequently display different tumor regression outcomes, which indicates that TNM staging is not enough to accurately predict tumor residual. Therefore, novel tools are needed to further identify NPC patients who may have residual lesions before treatment. Radiomics serves as a scientific approach for quantitatively describing the characteristics of medical image attributes [7]. Several studies have shown that radiomics characteristics derived from refined imaging data are effective imaging biomarkers for predicting therapy effect and prognosis in malignant tumor [8–10]. A radiomics model was established to calculate the individual risk value. The MRI data is most widely used for diagnosing and staging NPC [11, 12]. MRI can provide high‐quality tissue contrast information and is beneficial for the accurate delineation of tumor edges. Therefore, MRI was used to assess whether there was primary tumor residual after radiotherapy for NPC in the present research. Some scholars have reported that magnetic resonance radiomics is very important in the differential diagnosis of NPC, predicting the side effects of radiotherapy, and predicting prognosis. However, there are few reports on the prediction of residual NPC after radiotherapy by magnetic resonance radiomics.

We are committed to building a nomogram model that combines MRI and clinical characteristics to predict primary tumor residual and assist in individualized therapy.

## 2. Materials and Methods

### 2.1. Patients

This research collected NPC case information from December 2017 to March 2023 including laboratory examination, MRI imaging, treatment method, and clinical features. According to the criteria, 200 NPC cases were ultimately enrolled. The inclusion criteria are as follows: (1) pathologically confirmed NPC, (2) no synchronous or metachronous malignant tumor, (3) excellent visualization quality without any sequence missing or incomplete, (4) patients who had not received treatment before MRI scan, including radiotherapy or chemotherapy, and (5) both before and postradiotherapy MRI examinations of the nasopharynx and neck were performed in our hospital. Demographic information and relevant clinical parameters, including age, gender, T/N/clinical stage, Epstein–Barr virus quantification, pathologic histology type, induction chemotherapy, simultaneous chemotherapy, family history of nasopharyngeal cancer, platelet count, skull base invasion, and parotid gland invasion, were collected. The TNM staging was determined according to the AJCC TNM classification. The included cases were randomly allocated into two cohorts using a 4:1 ratio, with 40 cases in the independent validation set. In radiomics studies, a large set of high‐dimensional quantitative imaging features is usually extracted during model development, after which feature selection and machine learning–based modeling are carried out. Under such circumstances, a relatively larger training cohort is methodologically recommended to ensure stable feature selection, reliable parameter estimation, and robust model construction, particularly when the overall sample size is limited. At the same time, maintaining an independent validation cohort remains essential for an unbiased assessment of model generalizability. This data partitioning strategy is consistent with previous radiomics studies [9, 13]. Baseline clinical variables were collected, as presented in Table 1.

**Table 1:** Demographic and clinical characteristics of patients in the training cohort and validation cohort.

Characteristic	Type	Training cohort (%)	Validation cohort (%)	*p* value
Gender	Male	112 (70)	28 (70)	0.58
	Female	48 (30)	12 (30)	
Age (years)	< 45	59 (36.9)	21 (52.5)	0.07
	≥ 45	101 (63.1)	19 (47.5)	
T stage	1	18 (11.2)	3 (7.5)	0.52
	2	38 (23.8)	10 (25)	
	3	67 (41.9)	21 (52.5)	
	4	37 (23.1)	6 (15)	
N stage	0	17 (10.6)	2 (5)	0.54
	1	46 (28.8)	10 (25)	
	2	59 (36.8)	19 (47.5)	
	3	38 (23.8)	9 (22.5)	
Overall stage	I	9 (5.6)	2 (5)	0.40
	II	20 (12.5)	2 (5)	
	III	68 (42.5)	22 (55)	
	IVa	63 (39.4)	14 (35)	
EBV DNA (copies/mL)	≤ 4000	138 (86.3)	35 (87.5)	0.84
	> 4000	22 (13.7)	5 (12.5)	
Induction chemotherapy	Yes	117 (73.1)	34 (85)	0.12
	No	43 (26.9)	6 (15)	
Simultaneous chemotherapy	Yes	97 (60.6)	28 (70)	0.27
	No	63 (39.4)	12 (30)	
Histology	WHO Type I	3 (1.9)	0 (0)	0.34
	WHO Type II	79 (49.4)	16 (40)	
	WHO Type III	78 (48.7)	24 (60)	
Family history of nasopharyngeal carcinoma	Yes	9 (5.6)	4 (10)	0.52
	No	151 (94.4)	36 (90)	
Platelet counts, ×109/L	≤ 255	79 (49.4)	22 (55)	0.52
	> 255	81 (50.6)	18 (45)	
Skull base invasion	Yes	82 (51.3)	21 (52.5)	0.89
	No	78 (48.7)	19 (47.5)	
Parotid gland invasion	Yes	23 (14.4)	5 (12.5)	0.76
	No	137 (85.6)	35 (87.5)	

### 2.2. Treatment

All patients enrolled received intensity‐modulated radiotherapy (IMRT) or volumetric modulated arc therapy (VMAT) technique in this study. The target delineation and volumes were in accordance with reference to ICRU reports No. 50 and No. 62. RT was performed with 6 MV photon irradiation. Concurrent chemotherapy comprised of cisplatin at a dose of 40 mg/m [2] every week during the radiotherapy period. All patients underwent MRI after the end of radiotherapy. The median follow‐up period was 48.0 months (range: 3.27–88.5), which was determined according to the date of RT completion and final follow‐up or death.

### 2.3. Estimation of Residual Tumor

The diagnostic MRI for assessing primary tumor residual was performed immediately after radiotherapy completion. This early time point is consistent with studies evaluating initial tumor response [6, 14] and distinguishes the endpoint from later recurrence. The presence of primary tumor residual requires the consent of two experienced imaging experts and two senior radiation oncology specialists. MRI‐based diagnostic criteria for residual primary tumors followed previous research [4, 5] and were defined as follows: (1) Residual disease within the nasopharynx or elsewhere in the soft tissues exhibits three characteristic features: low signal on T1‐weighted sequences, enhancement after the injection of Gd‐DTPA, and high signal on T2‐weighted sequences. (2) Residual tumor involving the skull base presents with two features: destruction of the skull base bone together with soft tissue and no reduction—or even an increase—in the scope of bone enhancement compared with pretreatment images as described previously [15, 16].

### 2.4. Radiomics Workflow

**Figure 1:**
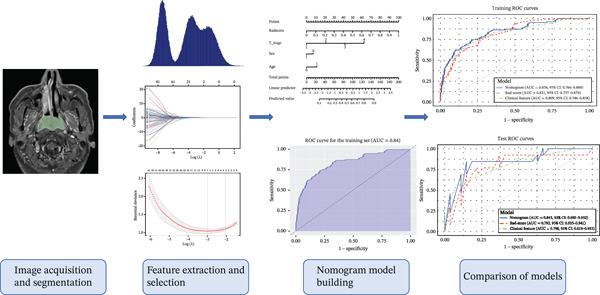
Radiomics workflow.

The flowchart of radiomics is presented in revised Figure 1, including (1) image acquisition and segmentation, (2) characteristic selection, (3) nomogram model building, and (4) comparison of models.

### 2.5. MRI Collection and Radiomics Characteristic Extraction

All participants underwent a baseline 1.5T MRI scan; we collected contrast‐enhanced T1‐weighted (CE‐T1WI) and T2WI sequences. The scanning range extended from a 2 cm area above the sella turcica, and the lower boundary extends to 2 cm below the clavicle′s inferior edge. The dose of 0.1 mmol/kg of body weight was intravenously injected at a speed of 2.5 mL/s. A senior radiation oncologist with extensive head‐and‐neck oncology experience outlined the tumor ROI on each slice of images; the ROI included the nasopharyngeal primary tumor, and each segmentation was validated by another specialist in radiation oncology to evaluate the MRI results. Any disagreements were treated based on consensus.

Before feature extraction, all the MRI images were normalized.

We extracted 109 radiomics features from seven VOIs across the two sequences (CE‐T1WI and T2WI) based on the radiomics toolkit PyRadiomics (https://pyradiomics.readthedocs.io/en/latest/index.html). The extracted features were as follows: first‐order features (*n* = 19), shape features (*n* = 15), gray‐level co‐occurrence matrix (GLCM) features (*n* = 24), gray‐level size zone matrix (GLSZM) features (*n* = 16), gray‐level run length matrix (GLRLM) features (*n* = 16), neighboring gray tone difference matrix (NGTDM) features (*n* = 5), and gray‐level dependence matrix (GLDM) features (*n* = 14). The LASSO was developed, and the results revealed substantial information on the predictor. The LASSO model is widely used for regression analysis involving complex predictors. It can precisely predict the occurrence, such as OS, in NPC. In the present research, we applied LASSO to identify features related to residual status from the collected MRI data.

### 2.6. Feature Selection and Nomogram Model Building and Validation

We utilized LASSO regression for feature selection of radiomics features to reduce dimensionality. Based on the trained LASSO regression model, feature coefficients were obtained, and a Rad‐score was calculated. A nomogram model was then developed using the Rad‐score and clinical features, which served as a regression model for prediction. This research used SMOTE for data preprocessing, which oversamples the minority class to balance the data. The evaluation metrics for the model included the AUC, accuracy (ACC), sensitivity (SEN), and specificity (SPE).

### 2.7. Statistical Analysis

The R 4.4.0 software was used to conduct the statistical analyses. The difference in clinical features between the two cohorts was tested by the chi‐squared test. Correlation analysis was applied to determine the association between variables. The *U* test was applied to analyze the association between clinical factors and primary tumor residual status in both cohorts. A multivariable regression model was applied to determine the predictors of primary tumor residual. A nomogram model was subsequently established based on integrating the Rad‐score with a multivariable regression model. The significance level was set at 0.05. The performance of the predictive nomogram was assessed via the index of AUC. To assess whether the differences in predictive effect among models were statistically significant, we compared the AUCs via the DeLong test. This nonparametric approach is suitable for comparing the AUCs of correlated ROC curves derived from the same dataset. The test was performed on the pROC package, with two‐sided *p* values reported. Pairwise comparisons were performed between the nomogram model, the radiomics‐only model (Rad‐score), and the model based solely on clinical variables. The decision curve analysis (DCA) was used to evaluate the influence of the nomogram model on clinical treatment decisions.

## 3. Results

### 3.1. Clinical Features of Cases

The 200 cases enrolled in the present research were grouped into training and validation cohorts. There were 140 male cases. There exists no obvious difference between the training cohort and the validation cohort on baseline features, like age, gender, clinical stage, Epstein–Barr virus quantification, pathologic histology type, induction chemotherapy, simultaneous chemotherapy, family history of NPC, platelet count, skull base invasion, and parotid gland invasion. The results were not significant (*p* > 0.05). Table 1 presents the clinical features of cases in both cohorts. In our study, among the 200 enrolled patients, 64 patients (32%) had primary tumor residual at the end of treatment, and 136 patients (68%) had no residual tumor. For the training cohort (*n* = 160), 51 cases (31.9%) had residual tumor, and 109 patients (68.1%) had no residual tumor. In the validation cohort (*n* = 40), 13 patients (32.5%) had residual tumor, and 27 patients (67.5%) had no residual tumor.

### 3.2. Radiomics Feature Extraction and Selection

**Figure 2:**
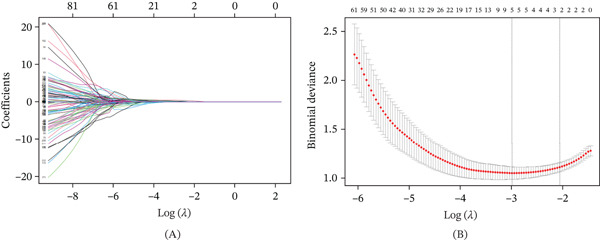
Selection of radiomics features for predicting primary tumor residual using the LASSO logistic regression model. (A) LASSO coefficient profiles of the radiomics features. (B) The cross‐validation curve.

**Figure 3:**
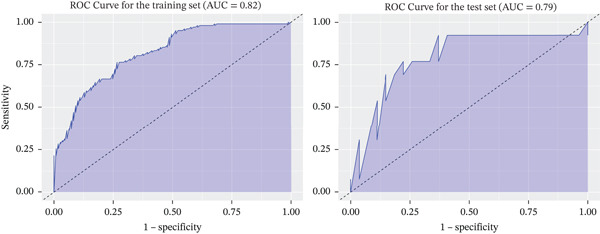
The ROC curve of the Rad‐score prediction model in the training set and validation set.

We employed the LASSO model to reduce the dimensionality of radiomics characteristics and to select those most predictive of primary tumor residual in the training cohort (Figure 2). Ultimately, seven radiomics features and one intercept were included. The seven radiomics features were T1C_shape_LeastAxisLength, T1C_glrlm_Gray LevelNonUniformityNormalized, T1C_glrlm_GrayLevelVariance, T1C_glszm_GrayLevelNonUniformity, T1C_glszm_ZoneEntropy, T2_shape_Flatness, and T2_shape_Maximum2DDiameterSlice. Table 2 lists the coefficients of these parameters and their respective contributions to the prediction model. The patients′ Rad‐score was weighted by independent predictive parameters and coefficients. The predictive effect of the Rad‐score for primary tumor residual was evaluated with ROC curves. As shown in Figure 3, the AUCs of the Rad‐score in the training set and validation set were 0.82 and 0.79, respectively.

**Table 2:** The radiomics features selected from the LASSO regression model.

Number	Radiomics features	Coefficient
1	Intercept	−0.860632315809762
2	T1C_shape_LeastAxisLength	0.154920441228544
3	T1C_glrlm_GrayLevelNonUniformityNormalized	−0.00520709704796599
4	T1C_glrlm_GrayLevelVariance	1.01349723018011e − 09
5	T1C_glszm_GrayLevelNonUniformity	0.484835752698801
6	T1C_glszm_ZoneEntropy	0.198702856311872
7	T2_shape_Flatness	0.215859732379384
8	T2_shape_Maximum2DDiameterSlice	0.0714978194717992

*Note:* The “intercept” entry represents the regression constant term and is not a radiomics feature.

### 3.3. Nomogram Construction and Validation

**Figure 4:**
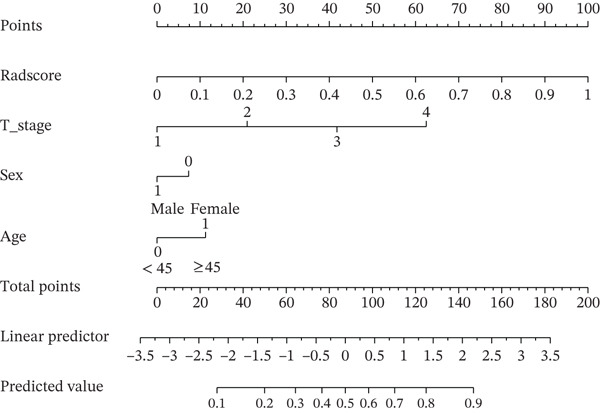
Development and validation of a predictive nomogram model for predicting primary tumor residual status.

**Figure 5:**
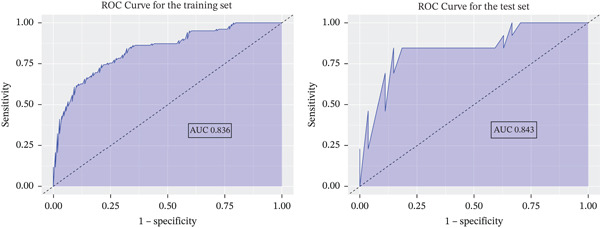
ROC for the nomogram in the training set and validation set.

**Figure 6:**
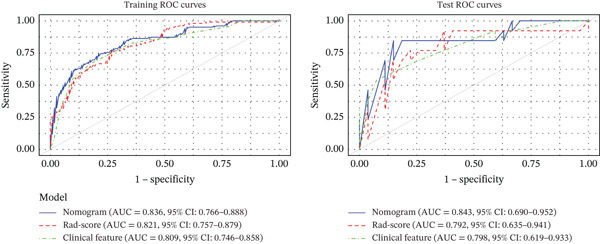
ROC curve comparison of the nomogram model, Rad‐score, and clinical feature in the training set and validation set.

We performed univariate and multivariate analyses to determine factors of primary tumor residual status. As shown in Table 3, univariate analysis results proved that the *p* value of T stage and overall stage was less than 0.1 in two cohorts, which were included in the multivariate regression model. Across both the two cohorts, the multivariate analysis result revealed that T stage was the only independent factor of primary tumor residual after radiotherapy in NPC patients (as shown in Table 4). At the same time, referring to previous literature reports, we incorporated gender and age into the nomogram model which was an easy‐to‐use tool to predict primary tumor residual status (Figure 4). As shown in Figure 5, the nomogram model achieved good predictive performance for post‐RT primary tumor residual for NPC patients in the training cohort, with an AUC of 0.836, and similarly good discrimination in the other cohort (AUC = 0.843). We then conducted the DeLong test to compare the AUCs of three models: the nomogram model, Rad‐score, and clinical model (Figure 6). It was found that, for the training cohort, the nomogram yielded an AUC of 0.836 and is obviously higher compared with that of the clinical model (AUC: 0.809, 95% CI: 0.746–0.858, *p* = 0.028) and the Rad‐score model (AUC: 0.821, 95% CI: 0.757–0.879, *p* = 0.046). Similarly, for validation cohort, the nomogram showed a highest AUC of 0.843 (95% CI: 0.690–0.952), with statistically superior performance compared with clinical model (AUC: 0.798, 95% CI: 0.619–0.933, *p* = 0.021) and the Rad‐score model (AUC: 0.792, 95% CI: 0.635–0.941, *p* = 0.034). These results demonstrated that combining clinical and radiomics characteristics significantly enhanced the model′s discriminatory ability, and it can be concluded that the nomogram consistently showed superior ability to identify primary tumor residues in the two cohorts.

**Table 3:** The association between clinicopathological characteristics and primary tumor residual status in NPC patients who received RT.

Variables	Training cohort (*n* = 160)		*p* value	Validation cohort (*n* = 40)		*p* value
	Local residual tumor	Nonlocal residual tumor		Local residual tumor	Nonlocal residual tumor	
Gender			0.319			0.024
Male	33	79		6	22	
Female	18	30		7	5	
Age (years)			0.325			0.223
< 45	16	43		5	16	
≥ 45	35	66		8	11	
T stage			< 0.001			0.028
1	0	18		0	3	
2	7	31		2	8	
3	17	50		6	15	
4	27	10		5	1	
N stage			0.103			0.621
0	1	16		0	2	
1	17	29		4	6	
2	21	38		7	12	
3	12	26		2	7	
Overall stage			0.003			0.086
I	0	9		0	2	
II	5	15		2	0	
III	16	52		5	17	
IVa	30	33		6	8	
EBV DNA (copies/mL)			0.995			0.529
≤ 4000	44	94		12	23	
> 4000	7	15		1	4	
Induction chemotherapy			0.302			0.963
Yes	40	77		11	23	
No	11	32		2	4	
Simultaneous chemotherapy			0.708			0.942
Yes	32	65		9	19	
No	19	44		4	8	
Histology			0.225			0.892
WHO Type I	0	3		0	0	
WHO Type II	22	57		5	11	
WHO Type III	29	49		8	16	
Family history of nasopharyngeal carcinoma			0.524			0.437
Yes	2	7		2	2	
No	49	102		11	25	
Platelet counts (×10^9^/L)			0.461			0.441
≤ 255	23	56		6	16	
> 255	28	53		7	11	
Skull base invasion			< 0.001			0.147
Yes	41	41		9	12	
No	10	68		4	15	
Parotid gland invasion			0.024			0.529
Yes	12	11		1	4	
No	39	98		12	23	

**Table 4:** Multivariate analysis of factors associated with primary tumor residual status for NPC patients who received RT.

Variables	Training cohort (*n* = 160)		Validation cohort (*n* = 40)	
	OR (95% CI)	*p* value	OR (95% CI)	*p* value
T stage	6.060 (2.364–15.537)	< 0.001	14.699 (1.553–139.113)	0.019
Overall stage	0.608 (0.229–1.615)	0.318	0.187 (0.021–1.685)	0.135

### 3.4. Clinical Benefits of Nomogram

**Figure 7:**
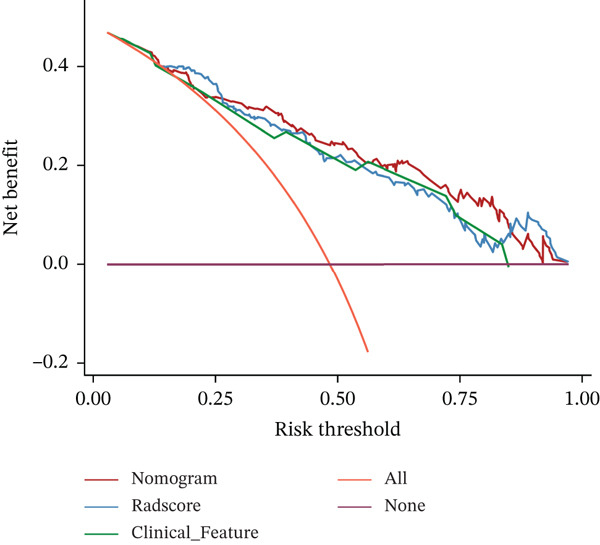
Decision curve analysis of the models.

The influence of the nomogram model on clinical treatment decision was analyzed by the DCA. As shown in Figure 7, the nomogram model showed better performance across most threshold ranges (0.26–0.86) with higher net benefit, indicating that it was the optimal decision tool within these thresholds.

## 4. Discussion

Radiotherapy remains a mainstay in the definitive management of NPC. In this research, we devised a radiomics nomogram that merged imaging‐derived signatures with clinical data and subsequently verified its performance. It provided a visual, personalized risk assessment tool, allowing clinicians to diagnose NPC with a high risk of primary tumor residual at a lower cost and earlier. In the two independent cohorts, the nomogram exhibited AUCs of 0.836 and 0.843, reflecting consistently high predictive power.

As a nascent image‐analytic technique, radiomics applies data characterization algorithms to convert CT, MRI, and PET/CT scans into quantitative characteristic data, including shape, wavelet feature intensity, and texture [17]. Subsequently, medical image information benefits disease diagnosis and the formulation of individualized treatment for patients. Nowadays, several investigators have analyzed the value of radiomics characteristics to predict the effect for cases with a variety of tumors [18–22]. Many researchers believe that the main reason why radiomics imaging can predict the efficacy of treatment is that the image information is derived from the entire tumor, so they can capture intratumoral heterogeneity more accurately. Incorporating radiomics features into predictive models can reveal tumor characteristics that are not always captured by clinical staging alone. Using pretreatment contrast‐enhanced CT scans from 226 esophageal cancer cases, Luo et al. [18] selected seven radiomics features and then built a prediction model that integrated these features with clinical staging. The model yielded AUCs of 0.807 for the validation set. Superior soft tissue characterization and multiplanar imaging capability are inherent advantages of MRI, enabling a clear and comprehensive visualization of both the normal and pathological anatomy of the nasopharynx. Therefore, in this study, MRI was used to evaluate the residual NPC after radiation. The MRI data in our study were acquired from a single vendor using a 1.5T scanner with relatively uniform sequence parameters. While this consistency minimizes technical variability, it may limit the model′s generalizability to external multicenter datasets. Differences in field strength, scanning protocols, or reconstruction algorithms could introduce radiomics feature distribution shifts, potentially affecting predictive performance. We chose to evaluate tumor residual after radiotherapy mainly based on previous studies [6, 14]. He et al. retrospectively compared the prognostic value of MRI detection residue at the end of radiotherapy; the presence of residual disease after RT was found to be independently and inversely associated with OS, LRFS, and DFS [6]. A similar result was obtained in this study [14]; the researchers believed that strengthening treatment may benefit NPC cases with poor outcomes after RT. Up to 5502 radiomics characteristics were derived by Wu and coworkers from the baseline MRI scans of 91 individuals diagnosed with locally advanced NPC [22]. They found that PLR and tumor volume independently predicted early treatment response. The clinical‐radiomics nomogram demonstrated robust predictive performance for early treatment response; however, the sample size of the present study was relatively limited. Our investigation included 200 NPC patients who received radiotherapy. From these data, we selected seven MRI characteristics and clinical variables to develop a nomogram that predicts post‐RT primary tumor residual. According to the predictive nomogram model, more timely and active treatment strategies are needed for patients with high residual risk.

In Table 1, in the baseline comparison of two groups, we included platelets mainly to take into account that, on the one hand, platelets are very important in promoting tumor cell growth, dissemination, and new blood vessel formation. Additionally, when circulating tumor cells associate with platelets in the bloodstream, they can enhance metastatic spread through direct interactions and the release of secreted bioactive proteins [23, 24]. On the other hand, studies have shown that platelets in NPC are associated with prognosis [25, 26]. However, univariate and multivariate factor analyses did not suggest that platelets were associated with primary tumor residual, so it is not included in the model.

Tumor staging remains a cornerstone prognostic indicator for cancer patients and directly informs therapeutic decision‐making. The T staging of NPC in the AJCC staging system represents the primary tumor of NPC, and its staging criteria primarily take into account both how far the tumor has spread and how deeply it has invaded adjacent anatomical structures. A more advanced T stage usually indicates a greater tumor load, which in turn leads to the increase of tumor hypoxia, the increase of clonal tumor cells, and the increase of radiotherapy resistance, as well as possible alterations in intercellular communication modes [27]. Previous investigations have shown that an advanced T stage is significantly associated with an increased risk of residual tumor [28, 29]. We further found a strongly significant relation in pretreatment T stage and primary tumor residual (*p* < 0.001 in two cohorts). The model analysis indicated that pretreatment T stage emerged as the sole independent predictor of primary tumor residual. Although the T stage is a strong predictor, incorporating radiomics features may offer more precise predictions by considering tumor differences, which are not always measured by clinical staging alone. The radiomics features also play a critical role. At the same time, while not significant in multivariate analysis, age and sex were maintained in the model for their recognized clinical relevance in NPC. Their inclusion improved model calibration and clinical net benefit and enhances the model′s interpretability as a practical decision‐making tool. Retaining these variables also supports future external validation in diverse populations. As a matter of common knowledge, advancing age is generally accompanied by a decline in performance status and an increased prevalence of comorbidities, reducing the treatment tolerance rate. Previous researches have demonstrated that age is correlated with the effectiveness and prognosis of treatment for NPC [30–33]. In the present study, the cutoff of 45 years was chosen based on previous literature and clinical relevance [34, 35]. We found that patients over 45 years of age are more likely to have primary tumor residues than patients under 45 years of age, which may be related to lower tolerance to treatment. In addition, a number of studies have shown that being male is a poor prognostic factor for NPC [34, 36–38], mainly because male patients are more likely to smoke than female patients. The study reported that smoking before treatment independently predicts poorer prognosis in NPC, including increased risks of death, disease progression, local recurrence, and distant metastasis [36]. But this study got a less consistent result; the findings of this study revealed that female patients exhibit a greater susceptibility to primary tumor residual, which is often associated with a less favorable prognosis. The reason why we considered this situation was that male patients with T3 and T4 accounted for 41.25%, whereas female patients accounted for 79.16% in the training cohort. However, some other variables, such as radiation dose, chemotherapy, or EBV DNA, may affect residual risk, and we did not include them in the model. The main reasons are as follows: On the one hand, in the article, we performed univariate and multivariate analyses of chemotherapy (simultaneous chemotherapy and induction chemotherapy) and EB virus quantification in the two sets. The finding demonstrated that they were not independent prognostic factors for primary tumor residual, so they were not included in the model. On the other hand, the level of radiation delivered is indeed a critical factor that affects the tumor residual in NPC. However, in this study, because this is a retrospective analysis, the detailed radiotherapy dose records of a few patients are incomplete or missing, and we cannot reliably obtain and standardize these dosimetric parameters in all enrolled patients. To guarantee the model′s robustness and reproducibility while preventing bias from data interpolation, we made a difficult decision not to include radiotherapy dose information in the initial model. In future prospective studies and model optimization, we will consider detailed radiotherapy dose records (such as total dose, fractional dose, and dose delivered to organs at risk) as key variables and devote ourselves to developing more comprehensive and accurate prediction tools to better serve the individualized treatment of NPC. Our nomogram obtained an AUC of 0.843 in the validation cohort, confirming its discriminative ability. Hence, in comparing the nomogram with T staging and clinical parameters, DCA demonstrated that the combined model offered superior clinical utility for predicting residual primary tumor in NPC.

We ultimately incorporated seven radiomics features into our nomogram model, comprising three shape descriptors (T1C_shape_LeastAxisLength, T2_shape_Flatness, and T2_shape_Maximum2DDiameterSlice) and four texture‐based metrics derived from GLRLM and GLSZM matrices (T1C_glrlm_GrayLevelNonUniformityNormalized, T1C_glrlm_GrayLevelVariance, T1C_glszm_GrayLevelNonUniformity, and T1C_glszm_ZoneEntropy). These features may capture tumor characteristics that are not fully captured by clinical staging alone, and their biological significance in relation to radioresistance mechanisms warrants systematic discussion. The shape features reflect the physical geometry and spatial extent of the tumor. LeastAxisLength represents the shortest axis of the three‐dimensional tumor volume, Maximum2DDiameterSlice quantifies the largest cross‐sectional diameter, and Flatness describes the degree to which the tumor shape is flattened (i.e., less compact in one dimension). Tumors with larger dimensions and less compact geometry typically indicate greater tumor burden and more aggressive growth patterns [39]. From a radiobiological perspective, larger tumors are more likely to contain hypoxic regions due to outgrowth of the existing vasculature and inadequate oxygen diffusion [40]. Hypoxia is a well‐established mechanism of radioresistance; the cytotoxic effect of ionizing radiation is largely oxygen‐dependent, as molecular oxygen is needed to generate ROS and to cause irreparable DNA structure breaks [41, 42]. Therefore, shape features may be indirectly associated with hypoxia‐related radioresistance by reflecting tumor burden and geometric complexity. The four texture features quantify the heterogeneity of signal intensity distribution within the tumor on CE‐T1WI. GrayLevelNonUniformity and GrayLevelNonUniformityNormalized measure the variability of gray‐level intensities, GrayLevelVariance captures the dispersion of intensity values, and ZoneEntropy reflects the randomness and complexity of spatial signal patterns [43]. Higher values of these metrics indicate more heterogeneous intratumoral architecture. Such imaging heterogeneity has been shown to correlate with underlying pathological and biological heterogeneity, including variations in cellular density, necrosis, fibrosis, and microvascular distribution [44, 45]. Importantly, there is growing recognition that intratumoral heterogeneity represents a hallmark of resistance to treatment, reflecting the coexistence of tumor cell subclones with differential radiosensitivity [46]. Heterogeneous tumors are more likely to harbor resistant subpopulations that survive radiotherapy and contribute to residual disease. The biological rationale for CE‐T1WI‐derived features is well established. Notably, four of the seven selected features were derived from CE‐T1WI. This predominance is biologically plausible, as gadolinium‐based contrast enhancement is impacted by microvascular perfusion at the time of acquisition [47]. Although CE‐T1WI is not a direct quantitative perfusion measure, the enhancement patterns may partially reflect tumor vascularity and blood supply status, which are important determinants of oxygen delivery to tumor cells and consequently influence radiosensitivity. Poorly enhanced tumor regions may represent areas with compromised vascular supply and potential hypoxic microenvironments associated with inherent radioresistance, although this remains an indirect inference from routine contrast‐enhanced imaging. Thus, texture features extracted from CE‐T1WI may capture vascular‐related biological information that is relevant to treatment response prediction. Notably, while the T stage remains the most significant clinical predictor, it represents a relatively coarse estimate of tumor burden based on anatomical extent. Radiomics features, by contrast, extract voxel‐level information from the entire tumor volume and can capture biologically relevant variations in tissue composition. This suggests that radiomics complements clinical staging by integrating tumor phenotype with spatial complexity, thereby enhancing individualized risk stratification. Our findings echo previous reports that radiomics is effective in predicting treatment response and posttreatment status [17, 48]. Integrating these features into the nomogram provides a noninvasive, imaging‐based biomarker framework, thereby enabling clinicians to identify high‐risk patients for residual disease and to tailor follow‐up or adjuvant strategies accordingly.

Recent comprehensive reviews systematically summarized the use of deep learning for NPC [49, 50]. Most models are designed to predict long‐term outcomes: OS, PFS, or distant metastasis; there is increasing interest in predicting short‐term treatment response. They also stressed persistent issues: protocol standardization, multicenter external validation, and the added value of imaging‐clinical data fusion for bolstering model performance and practical usefulness. Consistent with prior studies, our data underscore the clinical relevance of radiomics analysis in NPC. Compared with previous radiomics studies that predominantly focused on long‐term prognosis [9, 51, 52], our model specifically targets the clinically relevant endpoint of primary tumor residual immediately after radiotherapy. Early identification of residual disease enables timely intervention, such as adjuvant therapy or intensified follow‐up, which may improve patient outcomes. Although deep learning models have shown outstanding results in some imaging tasks, they often require large sample sizes and suffer from limited interpretability, which hinders their clinical adoption. In contrast, our nomogram integrates radiomics features with clinical variables in a transparent, regression‐based framework, offering a balance between predictive ACC and clinical interpretability. This makes it more accessible for routine practice. Furthermore, before widespread clinical implementation, large, multicenter external validation is a necessity. Future studies should also explore the combination of deep learning characteristics to further enhance model performance while maintaining interpretability.

## 5. Limitations

Certain limitations need to be noted regarding this research. Firstly, this retrospective study was conducted at a single institution in an endemic area. Secondly, the absence of detailed, standardized radiotherapy dosimetric parameters in our predictive model is clinically significant, as the actual delivered radiation dose and its distribution are fundamental biological determinants of tumor residual. Furthermore, we did not incorporate detailed indicators of chemotherapy sensitivity, such as cycles of induction chemotherapy regimens and the grade of acute toxicity. The absence of radiotherapy dosimetric data and chemotherapy sensitivity likely limits the model′s applicability in clinical practice, where treatment planning is highly individualized. Next, prospective and large sample radiomics studies which include comprehensive radiotherapy dosimetry and chemotherapy data should be carried out in both epidemic regions and nonepidemic regions of NPC to improve model efficiency and stability, prove its actual benefits, and promote the promotion and application of the model.

## 6. Conclusion

In conclusion, this research constructed and validated a nomogram model to predict primary tumor residual in patients with NPC after radiotherapy based on radiomics data and clinical variables. In clinical practice, the model allowed for early screening of patients who are susceptible to tumor residual, providing a novel and cost‐effective tool to guide timely intervention and individual treatment strategies for those patients.

## Author Contributions

Pingyan Liao and Min Zeng contributed equally to this work and should be considered co‐first authors.

## Funding

This work was supported by the Zhongshan Science and Technology Bureau (10.13039/501100017291; 2023B1067 and 2024B3046) and the Zhongshan Health Bureau (20231A020155).

## Conflicts of Interest

The study was supported by Zhongshan City Special Scientific Research Project for the Inheritance, Innovation, and Development of Traditional Chinese Medicine (NO.2024B3046) ,Zhongshan Medical Scientific Research Projects(NO.20231A020155) And The First Batch of Social Welfare and Basic Research Projects (General Medical and Health Projects) of Zhongshan City in 2023(NO. 2023B1067) .

## Data Availability

Research data are not shared.
